# Supplementary material for: Imbalance in the blood antioxidant system in growth hormone-deficient children before and after 1 year of recombinant growth hormone therapy
Source: PeerJ. 2015 Jun 25;3:e1055. doi: 10.7717/peerj.1055 (PMC4485705; doi:10.7717/peerj.1055)
Supplement: Supplemental Information 1 [file peerj-03-1055-s001.pdf]

ИНФОРМАЦИЯ ДЛЯ ПАЦИЕНТА И ИНФОРМИРОВАННОЕ СОГЛАСИЕ

для участия в клиническом исследовании

Врач-исследователь – к.м.н. Панкратова М.С. (тел: 89037294459, e-mail: ms.pankratova@gmail.com)

Тема исследования: ««Антиоксидантная система крови и функциональное состояние эритроцитов при нарушении секреции гормона роста».

Цель работы: Целью исследования является изучение системного влияния дефицита гормона роста и терапии препаратами рекомбинантного гормона роста на метаболизм организма ребенка на примере антиоксидантной системы крови и морфо-функционального состояния эритроцитов у детей с соматотропной недостаточностью.

В данном исследовании будут принимать участие пациенты с дефицитом гормона роста в возрасте от 3 до 12 лет до начала и на фоне терапии гормоном роста.

Если пациент не достиг 15 летнего возраста, согласие на участие в исследовании подписывает его законный представитель.

Уважаемый пациент (законный представитель ребенка), Вам (Вашему ребенку) предлагается участие в данном исследовании. О порядке его проведения Вам подробно расскажет врач-исследователь. Информация об исследовании представлена также в настоящей форме согласия. После того как Вы ознакомитесь с информацией, у Вас будет возможность задать врачу-исследователю или другому сотруднику исследовательского центра возникшие вопросы и получить дополнительные разъяснения. Если Вы примете решение участвовать в предложенном исследовании, то после того как вы получите ответы на все интересующие вопросы, Вам будет предложено письменно подтвердить свое согласие, поставив подпись на последней странице данной формы. Никакие процедуры исследования не могут быть проведены до тех пор, пока Вы не подпишете форму информированного согласия. Ваше участие в данной научно-исследовательской работе является добровольным. В любое время Вы можете принять решение о прекращении участия в исследовании. Ваше решение прекратить участие в исследовании не повлияет на порядок предоставления Вам (вашему ребенку) медицинской помощи и отношение к Вам (вашему ребенку) сотрудников исследовательского центра.

Информация об исследовании

Низкорослостью страдает около 2% населения, в том числе и в России. Одна из наиболее частых причин низкорослости - это соматотропная недостаточность. В течение последних 30 лет накоплен опыт успешного применения препаратов рекомбинантного гормона роста при различных

формах низкорослости. Однако несколько в стороне остается вопрос насколько эффективным является кровоснабжение органов и тканей у детей в случае ускорения роста всего организма и достаточно ли развивается кровеносная система, чтобы обеспечить приток кислорода ко всем органам и тканям.

Изменение кислородного статуса организма может привести к серьезным изменениям в организме, в частности развития окислительного стресса и др., а с другой стороны может являться маркером процессов происходящих в организме.

В случае увеличения скорости роста организма также наблюдается и ускорение метаболических процессов в организме, что в сочетании с изменением кислородного статуса может привести к развитию различного рода стрессов, в том числе и окислительного. Оценивая активность антиоксидантной системы крови можно судить о повреждающем действии этих форм на организм.

Вам предлагается принять участие в данном исследовании, в ходе которого будет проведено исследование функционального состояния гемоглобина и оценка его кислород-связывающих и кислород-транспортных свойств, а также функционального состояния антиоксидантной системы крови и уровня оксидантного стресса организма в условиях дефицита гормона роста, а также в условиях продолжительной терапии гормоном роста. На основании полученных результатов можно будет сделать заключение об общем состоянии организма и наличии гипоксии. В связи с исследованием Вашему ребенку будет произведен забор крови из вены в 1 пробирку объемом 4 мл. Лечение Вашего ребенка не будет связано с данным исследованием и будет соответствовать стандартам лечения для установленного Вам диагноза.

Возможные риски, побочные реакции. Возможные риски в ходе исследования минимальны и могут быть обусловлены процедурой забора крови. Забор крови может производиться натощак. Перед проведением процедуры необходимо предупредить врача-исследователя о ранее перенесённых осложнениях при проведении венепункции или о наличии гематологических заболеваний. Взятие образцов крови может сопровождаться небольшими болевыми ощущениями или осложниться образованием паравазальной гематомы (синяк), крайне редко - обмороком.

Конфиденциальность. Для защиты личной информации и обеспечения конфиденциальности записей будут предприниматься все необходимые меры, однако, полная конфиденциальность гарантирована не будет, т.к. результаты исследования могут быть опубликованы в медицинской литературе или журналах и представлены на научных конференциях, а также использованы в образовательных целях. Никакая личная информация в материалах, предназначенных для обучения или презентации широкому кругу лиц, раскрыта не будет.

Вопросы. При возникновении вопросов Вы можете обратиться к врачу-исследователю Панкратовой Марии Станиславовне

Подписи. Одна копия данного документа будет храниться в исследовательском центре вместе с записями, касающимися исследования. Вторая копия будет храниться у Вас. Третья копия будет храниться вместе с Вашими медицинскими записями в исследовательском центре.

В рамках исследования экспериментальные лечебные методики применяться не будут. Ваше участие в этом исследовании и использование Ваших данных является добровольным.

ИНФОРМИРОВАННОЕ СОГЛАСИЕ

Пациент или его законный представитель (в случае, если пациент не достиг 15 летнего возраста) ____

1. Я прочитал данный документ, содержащий информацию для пациента и форму информированного согласия на участие в исследовании. Мне объяснили сущность, цель и ожидаемые результаты исследования, а так же то, что я буду делать. Я имел возможность задать все интересующие меня вопросы и удовлетворён ответами на заданные мной вопросы.
2. Я подтвердил своё добровольное согласие на участие в клиническом исследовании. Я согласен полностью сотрудничать с исследователем.
3. Я даю согласие на то, чтобы врач проводил сбор образцов крови, и информации обо мне, при наличии показаний направлял меня на дополнительные инструментальные обследования.
4. Я даю согласие на возможность использования данных, полученных в результате клинического исследования, в научных целях.
5. Я даю согласие на проверку сведений, содержащихся в моей истории болезни, представителям органов здравоохранения, Этического комитета.
6. Я получил оригинал подписанного и датированного информированного согласия.

Подпись пациента ___________________________ Дата ___________

Подпись доверенного лица _______________________ Дата ___________

Подпись исследователя ___________________________ Дата ___________
