# Supplementary material for: Imbalance in the blood antioxidant system in growth hormone-deficient children before and after 1 year of recombinant growth hormone therapy
Source: PeerJ. 2015 Jun 25;3:e1055. doi: 10.7717/peerj.1055 (PMC4485705; doi:10.7717/peerj.1055)
Supplement: Table S1 [file peerj-03-1055-s002.docx]

| Control | | | | | | | | | | | | | |  |  |  |  |  |  |  |  |
| --- | --- | --- | --- | --- | --- | --- | --- | --- | --- | --- | --- | --- | --- | --- | --- | --- | --- | --- | --- | --- | --- |
| Patients | | Total antioxidant capacity of plasma | | Non-Protein Thiols | | Superoxide Dismutase (SOD) | | Catalase | | Ceruloplasmin | | age | |  |  |  |  |  |  |  |  |
|  | | μmol/L. | | µmol/ml | | Units/gHb | | k/gHb | | µkg/mL | | years | |  |  |  |  |  |  |  |  |
| 1 | | 820,7282913 | | 1,311 | | 18,61631764 | | 181,1783476 | | 524,7058824 | | 9,9 | |  |  |  |  |  |  |  |  |
| 2 | | 1084,033613 | | 0,8208 | | 17,92637956 | | 237,4578681 | | 457,6470588 | | 6,3 | |  |  |  |  |  |  |  |  |
| 3 | | 1078,431373 | | 0,741 | | 14,38334462 | | 190,4527702 | | 481,1764706 | | 9,5 | |  |  |  |  |  |  |  |  |
| 4 | | 663,8655462 | | 0,9633 | | 13,75146277 | | 240,8457565 | | 483,5294118 | | 10,5 | |  |  |  |  |  |  |  |  |
| 5 | | 588,2352941 | | 0,9348 | | 13,78115553 | | 205,7544536 | | 542,3529412 | | 9,1 | |  |  |  |  |  |  |  |  |
| 6 | | 686,2745098 | | 0,7296 | | 11,03973233 | | 221,157121 | | 412,9411765 | | 9,5 | |  |  |  |  |  |  |  |  |
| 7 | | 655,4621849 | | 0,6897 | | 10,14216504 | | 177,7190094 | | 487,0588235 | | 11 | |  |  |  |  |  |  |  |  |
| 8 | | 680,67 | | 0,969 | | 9,269330704 | | 212,9112602 | | 624,7058824 | | 10,2 | |  |  |  |  |  |  |  |  |
| 9 | | 470,5882353 | | 0,8322 | | 18,28975546 | | 219,3687885 | | 472,9411765 | | 7,5 | |  |  |  |  |  |  |  |  |
| 10 | | 697,4789916 | | 1,1172 | | 18,35699721 | | 307,3280109 | | 610,5882353 | | 10,2 | |  |  |  |  |  |  |  |  |
| 11 | | 563,0252101 | | 0,969 | | 14,26581608 | | 181,698056 | | 604,7058824 | | 9 | |  |  |  |  |  |  |  |  |
|  | |  | |  | |  | |  | |  | |  | |  |  |  |  |  |  |  |  |
| mean | | 726,254138 | | 0,916145455 | | 14,52931427 | | 215,9883129 | | 518,3957219 | | 9,336364 | |  |  |  |  |  |  |  |  |
| SD | | 196,3227406 | | 0,183679631 | | 3,426119441 | | 37,52103142 | | 69,51591932 | | 1,36914 | |  |  |  |  |  |  |  |  |
| SE | | 59,19397594 | | 0,055381906 | | 1,033021601 | | 11,31310119 | | 20,95999497 | | 0,412814 | |  |  |  |  |  |  |  |  |
| min | | 470,5882353 | | 0,6897 | | 9,269330704 | | 177,7190094 | | 412,9411765 | | 6,3 | |  |  |  |  |  |  |  |  |
| max | | 1084,033613 | | 1,311 | | 18,61631764 | | 307,3280109 | | 624,7058824 | | 11 | |  |  |  |  |  |  |  |  |
|  | |  | |  | |  | |  | |  | |  | |  |  |  |  |  |  |  |  |
|  | |  | |  | |  | |  | |  | |  | |  |  |  |  |  |  |  |  |
| Patients | Total antioxidant capacity of plasma | | Non-Protein Thiols | | Superoxide Dismutase | | Catalase | | Ceruloplasmin | | age | Height | Weight | | Height velocity | Height SDS | Velocity of height SDS | IGF-1 | IGFBP-3 | IGF-1 SDS | IGFBP-3 SDS |
|  | μmol/L | | µmol/ml | | Units/gHb | | k/gHb | | µkg/mL | | years | cm | kg | | cm/years |  |  | nMol/L | nMol/L |  |  |
| Before Treatment | | | | | | | | | | | | | | | | | | | | | |
| 1 | 389,355742 | | 0,798 | | 21,63296 | | 177,0872 | | 509,4118 | | 3,9 | 84,6 | 12,1 | | 3,98 | -3,78 | -2,78 | 3,2 | 73 | -3,90782 | -0,74575 |
| 2 | 745,098039 | | 0,627 | | 16,65905 | | 210,6439 | | 370,5882 | | 8,8 | 103,4 | 15,2 | | 4,14 | -4,57 | -1,72 | 3,2 | 41,7 | -5,06769 | -4,48102 |
| 3 | 756,302521 | | 0,1425 | | 31,71732 | | 272,2167 | | 724,7059 | | 3,35 | 84 | 12,1 | | 3,3 | -3,38 | -3,8 | 3,2 | 17,4 | -3,77763 | -7,73047 |
| 4 | 638,655462 | | 0,6327 | | 11,39544 | | 325,1478 | | 602,3529 | | 4,2 | 92,1 | 13,6 | | 3,63 | -2,14 | -3,06 | 14,6 | 11,3 | 0,822356 | -10,0332 |
| 5 | 344,537815 | | 0,8721 | | 15,23204 | | 287,9215 | | 635,2941 | | 5,7 | 98 | 17,5 | | 2,94 | -2,7 | -3,44 | 3,2 | 34,8 | -4,33389 | -4,76492 |
| 6 | 571,428571 | | 0,6441 | | 14,9226 | | 172,2959 | | 505,8824 | | 8,06 | 106,6 | 17,1 | | 2,45 | -3,32 | -4 | 10,5 | 118,3 | -1,13404 | 0,822519 |
| 7 | 504,201681 | | 0,8493 | | 15,49464 | | 194,0804 | | 524,7059 | | 7,6 | 109,7 | 17,2 | | 5,1 | -2,62 | -0,98 | 4,2 | 83,5 | -3,92348 | -0,81006 |
| 8 | 512,605042 | | 0,6897 | | 20,89165 | | 261,4856 | | 588,2353 | | 9,07 | 112,4 | 24,8 | | 1,8 | -3,4 | -5,8 | 10,5 | 101,1 | -1,37312 | -0,15364 |
| 9 | 663,865546 | | 0,6555 | | 22,22515 | | 106,1838 | | 577,1591 | | 7,7 | 94,1 | 12,2 | | 4,4 | -5,34 | -1,17 | 3,2 | 27,8 | -4,80731 | -6,26987 |
| 10 | 378,151261 | | 0,97 | | 20 | | 165,5 | | 654,12 | | 4,36 | 88,6 | 13,2 | | 4,28 | -3,44 | -2,22 | 3,2 | 24,3 | -4,0167 | -6,27735 |
| 11 | 268,907563 | | 0,85 | | 16,4 | | 193,2 | | 698,82 | | 3,9 | 84,8 | 12 | | 1,07 | -4,5 | -6,9 | 4,2 | 38,2 | -3,04766 | -3,94916 |
|  |  | |  | |  | |  | |  | |  |  |  | |  |  |  |  |  |  |  |
| mean | 524,828113 | | 0,702809 | | 18,77917 | | 215,0694 | | 581,0251 | | 6,058182 | 96,20909 | 15,18182 | | 3,371818 | -3,56273 | -3,26091 | 5,745455 | 51,94545 | -3,14245 | -4,03572 |
| SD | 165,831406 | | 0,219705 | | 5,443215 | | 64,31855 | | 100,7770 | | 2,208696 | 10,48937 | 3,873969 | | 1,212665 | 0,942869 | 1,840187 | 4,089832 | 36,10909 | 1,82083 | 3,46809 |
| SE | 50,0004240 | | 0,066244 | | 1,641203 | | 19,39292 | | 30,38564 | | 0,665952 | 3,162688 | 1,168054 | | 0,365635 | 0,284288 | 0,554841 | 1,23314 | 10,88738 | 0,549 | 1,04567 |
| min | 268,907563 | | 0,1425 | | 11,39544 | | 106,1838 | | 370,5882 | | 3,35 | 84 | 12 | | 1,07 | -5,34 | -6,9 | 3,2 | 11,3 | -5,06769 | -10,0332 |
| max | 756,302521 | | 0,97 | | 31,71732 | | 325,1478 | | 724,7059 | | 9,07 | 112,4 | 24,8 | | 5,1 | -2,14 | -0,98 | 14,6 | 118,3 | 0,82236 | 0,82252 |
|  | | | | | | | | | | | | | | | | | | | | | |
|  | | | | | | | | | | | | | | | | | | | | | |
| After Treatment | | | | | | | | | | | | | | | | | | | | | |
| 1 | 728,291317 | | 0,8151 | | 24,61022 | | 246,3018 | | 456,4706 | | 4,9 | 94,7 | 13,2 | | 10,76 | -2,64 | 3,56 | 6,1 | 93,9 | -2,10389 | 0,302574 |
| 2 | 834,733894 | | 0,57 | | 18,3 | | 167,5714 | | 394,1 | | 9,8 | 115 | 20 | | 12,4 | -3,33 | 9,26 | 12 | 97,4 | -1,12354 | -0,4819 |
| 3 | 551,820728 | | 0,83 | | 16,5 | | 175,40 | | 520,0 | | 4,35 | 97,2 | 13,3 | | 14,15 | -1,38 | 5,98 | 4,2 | 80 | -3,15418 | -0,38149 |
| 4 | 871,148459 | | 0,96 | | 27,3 | | 239,28 | | 602,4 | | 5,2 | 107,4 | 14 | | 15,88 | -0,13 | 8,61 | 14,4 | 125,2 | 0,542018 | 1,666455 |
| 5 | 641,456583 | | 0,79 | | 11,2 | | 201,38 | | 608,2 | | 6,7 | 115,8 | 23 | | 18,95 | -0,27 | 13,83 | 7,2 | 52,2 | -2,00554 | -2,95637 |
| 6 | 683,473389 | | 0,55 | | 16,2 | | 117,17 | | 387,1 | | 9,06 | 113,9 | 19,3 | | 7,66 | -2,97 | 2,87 | 34,2 | 166,5 | 2,364405 | 2,316021 |
| 7 | 535,014006 | | 0,72 | | 22,8 | | 215,11 | | 463,5 | | 8,6 | 118,2 | 19,8 | | 9,32 | -1,87 | 4,73 | 24,9 | 128,7 | 1,469454 | 1,132904 |
| 8 | 591,036415 | | 0,61 | | 22,3 | | 188,42 | | 729,4 | | 10,07 | 126,9 | 30,58 | | 14,6 | -1,95 | 14,2 | 17,3 | 138,6 | -0,03039 | 1,209825 |
| 9 | 669,467787 | | 0,79 | | 15 | | 176,1 | | 517,6 | | 8,7 | 102,4 | 13 | | 8,52 | -4,65 | 3,78 | 5,5 | 66,1 | -3,33089 | -2,18267 |
| 10 | 551,820728 | | 0,95 | | 18,6 | | 127,68 | | 484,7 | | 5,36 | 103,1 | 16,5 | | 14,55 | -1,53 | 7,63 | 7,6 | 55,6 | -1,51733 | -2,38021 |
| 11 | 577,030812 | | 0,98 | | 22,8 | | 236,9 | | 672,94 | | 4,9 | 93,3 | 13,4 | | 10,2 | -3,05 | 7,4 | 14,9 | 88,1 | 0,744668 | 0,006906 |
|  |  | |  | |  | |  | |  | |  |  |  | |  |  |  |  |  |  |  |
| mean | 657,754011 | | 0,778373 | | 19,59580 | | 190,1184 | | 530,5812 | | 7,049091 | 107,9909 | 17,82545 | | 12,45364 | -2,16091 | 7,440909 | 13,48182 | 99,3 | -0,74047 | -0,1589 |
| SD | 114,619016 | | 0,153903 | | 4,766148 | | 43,19516 | | 110,7104 | | 2,21865 | 10,82243 | 5,489911 | | 3,493483 | 1,344399 | 3,873749 | 9,260974 | 36,5466 | 1,88744 | 1,73923 |
| SE | 34,5591919 | | 0,046404 | | 1,437059 | | 13,02393 | | 33,38068 | | 0,668953 | 3,263109 | 1,655283 | | 1,053333 | 0,405355 | 1,167988 | 2,79231 | 11,0193 | 0,56909 | 0,5244 |
| min | 535,014006 | | 0,5472 | | 11,2 | | 117,1660 | | 387,0588 | | 4,35 | 93,3 | 13 | | 7,66 | -4,65 | 2,87 | 4,2 | 52,2 | -3,33089 | -2,95637 |
| max | 871,148459 | | 0,98 | | 27,25930 | | 246,3018 | | 729,4 | | 10,07 | 126,9 | 30,58 | | 18,95 | -0,13 | 14,2 | 34,2 | 166,5 | 2,3644 | 2,31602 |
